# The Best Are Barred

**Published:** 1896-12

**Authors:** 


The Best are Barred—It is to be presumed that the United States Government would want the very best medical officers, and would endeavor to secure the services of the most accomplished physicians and surgeons to be had, with which to recruit the medical corps of the army and navy. Indeed, as it is known that the examination necessary to be passed before one is admitted to the staff of either the army or navy, is very rigid, it would be inferred that only those of more than ordinary ability are wanted, or would be able to secure a position; and, as a matter of fact,—the examination is a fair test of professional attainments, and it is well enough that it is strict; but in consequence of the age-limit, which is ridicuously low,—the very kind of men sought to be enlisted in the service are excluded; *cœteris paribus.*

The limit in the Navy is 26 year, in the Army 28, and in the Marine Hospital service 29 years. That is to say, no physician over 26 years of age—no matter what his ability may be, will be permitted to apply for the examination for surgeon in the navy, and none over 28 years of age can enter the medical corps of the army;—nor those over 29 enter the Marine Hospital service as surgeon.

If the limit were reversed, and none *under* those ages were eligible, it would be better. As it is, few young men graduate under 21; and now that all the colleges are requiring three or four courses of study as condition to the degree, it is likely that in future, few or none will even graduate under 24 years of age. After graduating many serve two or more years as *interne* or resident physician in some hospital, and oftener than otherwise the more ambitious and better educated will want to take one or two courses of P. G. instruction; hence, by the time one is really qualified to pass the army and navy examination, he is past the age, and is cut off.

Really, the knowledge *how not to do it*, seems to be the chief accomplishment of the United States Government in many respects.

---

The JOURNAL greets its thousands of friends, and wishes them all a *Merry Christmas!* May prosperity and happiness attend every blessed doctor, worthy of the name, and may they gather in the ducats the coming year—to their hearts' content. Josh Billings said, "blessed is he who has his corns stepped on, for then he shall know how good it is to feel easy," (when it quits hurting). If we had no hard times and privations, we could not appreciate prosperity; and "spring," you know, "would be but gloomy weather, if we had nothing else but spring." So, as it often happens that a disappointment is a blessing in disguise, and often turns out to be the very best thing for one, that could have happened, let us regard the recent panics and money stringency, and hard times, metaphorically, in the light of a pet corn trodden on, and appreciate the fact that it has *quit hurting*.

It is only by comparison that we can appreciate any blessing. When the reaction comes—its coming now, they say; is in sight; —let us enjoy it the more for having suffered so long.

Yes: a *Merry* Christmas! and a *Happy* New Year! It is not "all of life to live," and it is not all of a doctor's life to practice. He is human, and enjoys a little relaxation, as do other folks. Let us, then, have a Merry Christmas; and after January 1st, buckle on, anew, the "harness," with the hope that it may be less galling, and start the year 1897, *with renewed hope*. Merry Christmas, doctor!

---

Dr. Hiram H. Darr, of Caldwell, Texas, died at his home, in that city, on the 22nd of November (ult.), aged 43. Dr. Darr was born in Caldwell county, Texas, April 4, 1853. His parents were natives of Tennessee and Virginia. He graduated with first honors in medicine at the Louisville, Ky., Medical College in February, 1875, and received the gold medal for general proficiency in all branches.

Dr. Darr was Vice President of the State Medical Association in 1884, and was a member of the American Medical Association and the American Public Health Association. He was a Knight Templar and a Mason.

---

Dr. Sessions in this issue has a paper on "Typho-Malarial Fever," which will interest our readers. He says, jestingly, that if a mixture of the malarial element will so modify the typhoid fever as it appears to do, it would be well to inoculate our typhoid cases with the malarial plasmodium as a curative measure. Our experience with what is called typho-malarial fever is, that it is about as obstinate, and as fatal as typhoid. We were under the impression that the medical profession had discarded the name—typho-malarial fever—repudiated it; and that all were agreed that what has been called typho-malarial fever heretofore, is typhoid, pure and simple,—only milder or—less severe; and that most of the doctors had adopted the plan of using quinine *as a means of diagnosis*. If a few doses, in the beginning, do not interrupt the remissions—they make a diagnosis of typhoid fever, and treat it (or let it run its course) accordingly.

